# Interleukin-6 Levels and Interleukin-6 rs1800795 Variant in Pediatric Familial Mediterranean Fever: Associations with Clinical Manifestations, Inflammatory Markers, and MEFV Mutation Status

**DOI:** 10.3390/diagnostics16172689

**Published:** 2026-08-23

**Authors:** Seyda Dogantan, Yasemin Oyaci, Adem Keskin, Fatima Ceren Tuncel, Sacide Pehlivan

**Affiliations:** 1 Institute of Graduate Studies in Health Sciences, Istanbul University, Istanbul 34390, Turkey; 2 Department of Pediatric Rheumatology, Başakşehir Çam ve Sakura City Hospital, Istanbul 34480, Turkey; 3 Department of Basic Sciences, Istanbul Nisantasi University, Istanbul 34481, Turkey; yaseminf@hotmail.com; 4 Department of Medical Biochemistry, Faculty of Medicine, Aydin Adnan Menderes University, Aydin 09010, Turkey; adem.keskin@adu.edu.tr; 5 Department of Medical Biology, Faculty of Medicine, Istanbul University, Istanbul 34390, Turkey; fatimaceren.tuncel@gmail.com (F.C.T.); sacide.pehlivan@istanbul.edu.tr (S.P.)

**Keywords:** Familial Mediterranean Fever, interleukin-6, *MEFV*, serum amyloid-A, C-reactive protein, symptom, erythrocyte sedimentation rate, *interleukin-6* rs1800795 variant, disease duration, ISSF score, annual attack frequency, colchicine-resistant

## Abstract

**Background/Objectives**: Interleukin-6 is a key proinflammatory cytokine involved in the pathophysiology of Familial Mediterranean Fever (FMF). This study aimed to evaluate interleukin-6 levels and the *interleukin-6* rs1800795 variant in pediatric FMF participants and to investigate their associations with clinical manifestations, laboratory findings, and *Mediterranean fever (MEFV)* mutation status. **Methods**: The research involved 69 pediatric FMF participants and 50 healthy children. Interleukin-6 levels, laboratory findings, and genotype distributions of the *interleukin-6* rs1800795 variant were compared between FMF and control groups. Moreover, the relationship between interleukin-6 levels and clinical findings, *MEFV* mutation status, and laboratory parameters were evaluated in the FMF group. **Results**: In the FMF group, interleukin-6, serum amyloid-A (SAA), white blood cell count (WBC), neutrophil, C-reactive protein (CRP), and erythrocyte sedimentation rate (ESR) levels were higher compared to the control group. In the FMF group, interleukin-6 levels showed a positive correlation with ISSF score, annual attack frequency, the number of concomitant symptoms, disease duration, SAA, CRP, and ESR. The allele and genotype distribution of the *interleukin-6* rs1800795 variant was similar to those in the control and FMF groups. In the FMF group, interleukin-6 values were higher in patients with abdominal pain, fever, chest pain, or arthralgia compared to those without these symptoms. Interleukin-6 levels were higher in both homozygous and compound heterozygous subgroups compared to the heterozygous subgroup. **Conclusions**: Elevated interleukin-6 levels in symptomatic pediatric FMF patients and those with homozygous or compound heterozygous *MEFV* mutations suggest that interleukin-6 may reflect both clinical disease severity and *MEFV* mutation status.

## 1. Introduction

FMF (Familial Mediterranean Fever) is the most common monogenic autoinflammatory illness globally, defined by repetitive, stereotypical fever episodes lasting less than three days, usually beginning in childhood. These episodes are frequently accompanied by abdominal symptoms, serositis, and a marked response to inflammation; while attacks resolve spontaneously, an increase in acute phase reactants is observed during the active period. The disease is particularly prevalent in populations of Mediterranean origin, and diagnosis is usually supported by demonstrating pathogenic variants in the *MEFV* gene, especially exon 10 [1,2]. While FMF is classically defined by intermittent episodes of fever and serosal inflammation, the disease is increasingly associated with ongoing low-grade inflammation and chronic inflammatory complications. The heightened inflammatory activity observed during attacks is reflected by increased acute-phase reactants, potentially resulting in enhanced oxidative stress and its related pathological effects [3].

Interleukin-6 plays an important role in the pathophysiology of autoinflammatory illnesses, characterized by the dysregulation of the innate immune system in the absence of antigen-specific T-cell responses or pathogenic autoantibodies. Unlike cytokines such as interleukin-1β and interleukin-18, which play a central role in the pathogenesis of FMF, interleukin-6 plays a role in potentiating the inflammation response and regulating the acute phase response. This suggests that interleukin-6 may be effective in disease development and could be considered a promising target for therapy [4]. Due to the potential role of interleukin-6 in the pathophysiology of FMF, anti-interleukin-6 therapy has emerged as a potential therapy option in individuals with colchicine-resistant or colchicine-intolerant FMF. Although current data are limited and follow-up periods are short, this therapy has been reported to show promising results in terms of both safety and efficacy in adult population with FMF [5].

Numerous research has suggested elevated serum interleukin-6 values in FMF patients, particularly during acute attacks. Furthermore, increased interleukin-6 concentrations have been observed even during non-attack periods, supporting the presence of persistent subclinical inflammation in FMF. High interleukin-6 levels have been associated with disease activity and inflammatory markers, suggesting that interleukin-6 may contribute to the chronic inflammatory burden of the disease [6,7,8]. However, current data are limited and unclear, particularly in pediatric populations, and the relationship between interleukin-6 levels, clinical manifestations, and genetic factors is not fully understood.

Interleukin-6 production and expression levels can be affected by variants in the transcriptional regulatory region of the *interleukin-6* gene [9]. Among the variants located in transcriptional regulatory region of the *interleukin-6* gene, the rs1800795 (-174 G>C) variant is one of the most extensively studied genetic variants. This variant has been the subject of several recent research in a wide range of illnesses, involving rheumatological illnesses such as vasculitis, osteoarthritis, and rheumatoid arthritis, as well as various cancers and diabetes mellitus. Because of its potential to influence interleukin-6 expression and inflammatory responses, the rs1800795 variant has been extensively investigated in terms of its association with disease susceptibility, disease severity, and clinical outcomes [9,10,11,12,13,14].

A recent study from Poland evaluated the association between the rs1800795 variant and clinical parameters in patients with periodontitis. No significant difference was observed in genotype or allele distributions between patients and healthy controls, and no significant association was found between rs1800795 genotypes and clinical parameters in the patient group [15]. In contrast, a recent study from Iran reported that the rs1800795 GG genotype and the G allele were associated with higher disease severity in patients with COVID-19. Furthermore, the GG genotype, along with INR, D-dimer, respiratory rate, and serum interleukin-6 levels, was identified as an independent predictor of COVID-19 mortality [16]. Consistent with these heterogeneous findings, a comprehensive meta-analysis involving 42,150 participants demonstrated that the functional effects of *interleukin-6* rs1800795 polymorphism may vary across different diseases, including diabetes mellitus, and may also differ according to the ethnicity of the populations studied, such as Asian, Caucasian, and mixed populations [17].

Although C-reactive protein (CRP), erythrocyte sedimentation rate (ESR), and especially serum amyloid A (SAA) are commonly used to assess inflammatory activity in FMF, these markers primarily reflect subphases of the acute phase response and may not fully reflect the clinical heterogeneity of the disease. In particular, identifying patients with higher inflammatory and clinical burdens, including those exhibiting more severe disease manifestations or exhibiting inadequate colchicine response, is clinically important in pediatric FMF. Therefore, investigating additional biomarkers that can provide complementary information to conventional inflammatory markers may contribute to the better characterization of the disease phenotype and inflammatory burden.

Interleukin-6 is of particular interest in this context, as it is a key subphase mediator of innate inflammatory signaling and contributes to the induction of the acute phase response. In FMF, dysregulated *MEFV*/pyrin inflammatory activation promotes inflammatory signaling, particularly via interleukin-1β and interleukin-18, which may subsequently contribute to increased interleukin-6 production. Therefore, interleukin-6 may establish a biologically significant link between *MEFV*-associated inflammatory activation and systemic inflammatory manifestations.

Evaluating interleukin-6 alongside conventional inflammatory markers and clinical features may help clarify whether interleukin-6 reflects differences in clinical inflammatory load beyond the expected increase during acute attacks. While elevated interleukin-6 concentrations during acute FMF attacks have been previously reported, the clinical significance of interleukin-6 in pediatric FMF is not fully characterized. Specifically, limited data exists regarding whether interleukin-6 levels are associated with the extent of clinical manifestations, disease severity, relapse frequency, colchicine resistance, and *MEFV* mutation status, as well as whether interleukin-6 can differentiate patients with more severe clinical phenotypes. Furthermore, the potential contribution of the *interleukin-6* rs1800795 variant to FMF susceptibility and circulating interleukin-6 concentrations has not been adequately investigated in pediatric populations.

Therefore, this study aimed to evaluate serum interleukin-6 levels and the *interleukin-6* rs1800795 variant in children with FMF and to investigate their associations with clinical manifestations, established inflammatory markers, disease severity, colchicine resistance, and *MEFV* mutation status. Additionally, receiver operating characteristic (ROC) curve analyses were performed to investigate the ability of interleukin-6 to differentiate clinically relevant disease phenotypes. By integrating biochemical, clinical, genetic, and diagnostic performance analyses, this study aimed to determine whether interleukin-6 can provide complementary information to characterize inflammatory burden and clinical heterogeneity in pediatric FMF.

## 2. Materials and Methods

### 2.1. Research Design

Prior to the commencement of the research, ethical approval was obtained from the Basaksehir Cam and Sakura City Hospital Local Ethics Committee (Approval Number = E-96317027-514.10-241609175). Accordingly, written informed consent was obtained from all kids and legal guardians before enrollment in the research, in accordance with the ethical principles of the Helsinki Declaration. The required sample size was calculated using G*Power version 3.1.9.7 (test family = *t*-tests, statistical test = Wilcoxon–Mann–Whitney test for two independent groups, one-way; effect size d = 0.65; probability of error α = 0.05; power = 0.95; distribution ratio N2/N1 = 1.38). The analysis showed that at least 65 patients and 47 controls were required.

### 2.2. Research Population

A total of 69 consecutive children diagnosed with FMF were recruited from the Pediatric Rheumatology Outpatient Clinic of Başakşehir Çam and Sakura City Hospital. These children were involved in the FMF group. The diagnosis of FMF was established using the PRINTO (Eurofever/Pediatric Rheumatology International Trials Organization) classification criteria. Diagnostic evaluation was further supported by the presence of compatible clinical findings according to the criteria of Tel-Hashomer and by genetic analysis of the *MEFV* gene when available [18,19]. Patients were classified as homozygous, heterozygous, or compound heterozygous according to their *MEFV* mutation status. Tel-Hashomer criteria were used as supportive diagnostic criteria, particularly in heterozygous patients. In addition, all heterozygous patients had a positive family history of FMF involving at least one first-degree relative (mother or father), supporting the clinical diagnosis. Thus, the diagnosis of FMF was based on the overall clinical evaluation together with genetic findings, rather than on genetic results alone.

Although recurrent fever is a common manifestation of FMF, it is not mandatory for diagnosis according to the Eurofever/PRINTO classification criteria. Patients without fever fulfilled the PRINTO classification criteria based on other characteristic clinical manifestations, including recurrent serositis (abdominal pain and/or chest pain), arthritis/arthralgia, supportive Tel-Hashomer criteria, and *MEFV* mutation analysis. The final diagnosis was confirmed by an experienced pediatric rheumatologist.

The mean age at diagnosis was 7.05 ± 4.06 years, and the mean disease duration was 50.18 ± 37.50 months. Peripheral venous blood samples were collected from all patients during acute FMF attacks. Patients with concomitant chronic inflammatory, autoimmune, infectious, malignant, or other systemic diseases were excluded. Clinical characteristics and laboratory findings of all patients were registered and evaluated.

All patients were receiving colchicine treatment at the time of blood sampling. The colchicine dose recorded in the study represents the current daily maintenance dose at the time of sample collection and is expressed in mg/kg/day according to each patient’s body weight. Colchicine treatment was prescribed and dosed by pediatric rheumatologists according to routine clinical practice.

The control group consisted of 50 healthy children from the same geographical region. To minimize potential confounding factors, health controls were selected to have comparable age and gender distributions to the FMF group.

### 2.3. Laboratory Analysis

All laboratory measurements, including interleukin-6, SAA, CRP, ESR, and complete blood count parameters, were performed using peripheral venous blood samples obtained during acute FMF attacks. An acute FMF attack was defined according to the Eurofever/PRINTO classification criteria and was confirmed by a pediatric rheumatologist based on the presence of typical clinical manifestations. Blood samples were obtained during the acute attack before laboratory analyses were performed.

### 2.4. Genotyping Analysis

Approximately 5 mL of peripheral venous blood specimen was provided from each kid. Genomic DNA was isolated from this specimen using the QIAamp DNA Blood Mini Kit (Qiagen GmbH, Hilden, Germany) following the manufacturer’s instructions. The *interleukin-6* rs1800795 (-174 G>C) variant was genotyped by PCR–RFLP (polymerase chain reaction–restriction fragment length polymorphism) analysis, as previously described [20,21,22].

The target region was amplified using the forward primer 5′-TGACTTCAGCTTTACTCTTTG-3′ and the reverse primer 5′-CTGATTGGAAACCTTATTAAG-3′. PCR amplification was performed under the following conditions: initial denaturation at 95 °C for 5 min, followed by 35 cycles of denaturation at 95 °C for 30 s, annealing at 52 °C for 30 s, and extension at 72 °C for 45 s, with a final extension at 72 °C for 5 min [22].

The PCR products were digested with the NlaIII restriction endonuclease. The resulting restriction fragments were separated by agarose gel electrophoresis and visualized under ultraviolet light for genotype determination. The GG genotype produced a single 168 bp fragment; the GC genotype produced 168, 119, and 49 bp fragments; and the CC genotype produced 119 and 49 bp fragments [22].

*MEFV* genotyping was performed using genomic DNA isolated from peripheral blood samples with the commercially available *MEFV* 15 Mutation Panel, *MEFV* MSQ 1.1 (HELİKS R&D and Biotechnology Inc., 34758, Ataşehir, Istanbul, Türkiye). based on DNA fragment analysis. The assay screened for the following *MEFV* variants: E148Q, P369S, F479L, M680I (G/A and G/C), M694V, M694I, I692del, K695R, V726A, A744S, and R761H. The assay was designed to detect these targeted *MEFV* variants and did not include full-gene sequencing or copy-number variant analysis.

Parental *MEFV* genotyping results were available for all patients. Segregation analysis demonstrated that all identified *MEFV* variants were inherited from one or both parents, and no de novo variants were identified. In patients with compound heterozygous genotypes, the parental origin of each variant was evaluated separately. The segregation results supported the molecular diagnosis in all patients.

### 2.5. Statistical Analysis

Statistical tests were performed using SPSS version 22.0 (IBM Corp., Armonk, NY, USA). Continuous variables were reported as mean ± standard deviation (SD), whereas categorical variables were reported as percentage (%) and number (n). Comparisons between two independent groups were performed using the Mann–Whitney U test, while comparisons among three or more subgroups were conducted using one-way test of variance (ANOVA) followed by the Dunnett T3 post hoc test when appropriate. Categorical data were compared using the chi-square analysis. In addition, ROC analysis was performed to evaluate the performance of interleukin-6 levels in discriminating some clinical and genetic characteristics. Correlations between data were examined using Spearman’s rank correlation test. A *p*-value < 0.05 was accepted as statistically important.

To control for potential confounding factors, multivariate linear regression analyses were performed for the main comparisons. Comparisons of interleukin-6 levels between FMF and control groups were adjusted for age and sex. Comparisons based on *MEFV* genotype and clinical symptom status were adjusted for age, sex, disease duration, and colchicine dose. Regression coefficients (B) are reported with 95% confidence intervals (95% CI). Bonferroni correction was applied for multiple pairwise comparisons where appropriate.

## 3. Results

### 3.1. Demographic Characteristics and Laboratory Parameters of the Study Groups

The research involved 69 pediatric participants aged 4–17 years diagnosed with FMF (FMF group). Additionally, 50 healthy kids aged 4–17 years were involved as a control group. The average age of the FMF group was 11.23 ± 4.26, while the average age of the control group was 12.72 ± 4.24. 57.97% (*n* = 40) of the FMF group were girls, compared to 58% (*n* = 29) in the control group. There was no important difference between the two groups in terms of average age and gender ratio (*p* = 0.061 and *p* = 0.997, respectively). Laboratory parameters for these two groups are provided in Table 1.

The FMF group had higher levels of interleukin-6, SAA, CRP, ESR, WBC, and neutrophil compared to the control group (Table 1). After adjustment for age and sex, FMF patients had interleukin-6 levels that were 38.99 pg/mL higher than those of healthy controls (adjusted mean difference (B): 38.99 pg/mL, 95% Confidence Interval (CI): 27.39–50.59; *p* < 0.001). To evaluate the relationships between interleukin-6 values, which showed statistically important differences between the two groups and other parameters found to be significant, a separate correlation analysis was conducted for each group. The results of the correlation analysis are provided in Table 2.

In the control group, interleukin-6 values demonstrated a positive correlation only with WBCs, while in the FMF group, interleukin-6 values demonstrated a positive correlation with SAA, CRP, and ESR values, but no significant relationship was found with WBC counts (Table 2).

### 3.2. Distribution of Interleukin-6 rs1800795 (-174 G>C) Variant

To evaluate the possible genetic basis for the difference in interleukin-6 levels detected between the two groups, the distributions of the *interleukin-6* rs1800795 (-174 G>C) gene variant were analyzed in the FMF and control groups. The results are provided in Table 3.

The genotype distributions of the *interleukin-6* rs1800795 variant were consistent with Hardy–Weinberg equilibrium in both the control group and the FMF group (Table 3). No statistically important difference was determined between the FMF and control groups in terms of genotype and allele distribution of the *interleukin-6* rs1800795 variant (Table 3).

### 3.3. Association of Interleukin-6 Levels with Clinical Characteristics in FMF Participants

In the FMF group, the mean disease duration was 50.18 ± 37.50 months, the mean colchicine dose was 0.03 mg/kg/day, the mean International Severity Scoring System for Familial Mediterranean Fever (ISSF) score was 1.49 ± 1.60, the mean annual attack frequency was 1.70 ± 1.39, and the mean number of concomitant symptoms was 2.7 ± 1.39. Disease duration (ρ = 0.256, *p* = 0.034), ISSF score (ρ = 0.575, *p* < 0.001), annual attack frequency (ρ = 0.461, *p* < 0.001), and the number of concomitant symptoms (ρ = 0.562, *p* < 0.001) were positively correlated with serum interleukin-6 levels. In contrast, no significant correlation was observed between colchicine dose and serum interleukin-6 levels (ρ = 0.128, *p* = 0.293). The results of comparing interleukin-6 levels according to the presence of symptoms, colchicine resistance, and ISSF severity categories in participants in the FMF group are presented in Table 4.

All patients without fever met the Eurofever/PRINTO classification criteria based on other characteristic clinical findings and underwent supportive *MEFV* mutation analysis; the diagnosis was confirmed by a pediatric rheumatologist.

In the FMF group, interleukin-6 values were significantly higher in participants with abdominal pain, fever, chest pain, or arthralgia compared with those without these symptoms (Table 4). In addition, participants with partial colchicine resistance had significantly higher interleukin-6 levels than those without colchicine resistance. Similarly, interleukin-6 levels increased according to ISSF severity categories and were significantly higher in patients with moderate disease severity than in those with mild disease severity.

The association between the number of clinical symptoms and serum interleukin-6 levels was evaluated using a multivariable linear regression model adjusted for age, sex, disease duration, and colchicine dose. The mean serum interleukin-6 levels were 21.44 ± 37.20 pg/mL in patients with one symptom (*n* = 11), 22.75 ± 29.05 pg/mL in those with two symptoms (*n* = 33), 51.68 ± 37.20 pg/mL in those with three symptoms (*n* = 4), and 82.26 ± 33.12 pg/mL in those with four symptoms (*n* = 21). After adjustment for potential confounders, serum interleukin-6 levels differed significantly according to the number of clinical symptoms (overall *p* < 0.001). Bonferroni-adjusted pairwise comparisons demonstrated that patients with four symptoms had significantly higher adjusted serum interleukin-6 levels than those with one symptom (B = 58.51 pg/mL, 95% CI: 26.99–90.04, *p* < 0.001) and those with two symptoms (B = 63.30 pg/mL, 95% CI: 38.42–88.18, *p* < 0.001). No significant differences were observed among the remaining pairwise comparisons (all *p* > 0.05).

### 3.4. Association Between MEFV Mutation Status and Interleukin-6 Levels

The distribution of *MEFV* gene mutations detected in the FMF group is provided in Table 5.

When the distribution of *MEFV* gene mutations was examined in the FMF group, compound heterozygous mutations were detected in 17 participants (24.64%), heterozygous mutations were detected in 36 participants (52.17%), and homozygous mutations were detected in 16 participants (23.19%). Patients were divided into subgroups according to these mutation statuses, and their interleukin-6 levels were compared (Table 6).

Interleukin-6 levels differed significantly among the subgroups defined according to *MEFV* mutation status (Table 6). Interleukin-6 levels were found to be higher in both compound heterozygous (B: 34.48 pg/mL, 95% CI: 7.53–61.44; *p* = 0.008) and homozygous (B: 50.21 pg/mL, 95% CI: 23.55–76.87; *p* < 0.001) subgroups compared to the heterozygous subgroup (Table 6). Conversely, there was no important difference in interleukin-6 values between the homozygous and compound heterozygous subgroups (*p* = 0.671) (Table 6).

Significant differences were observed among the *MEFV* mutation subgroups with respect to annual attack frequency and the distribution of colchicine resistance, whereas no significant difference was found in the age at diagnosis (Table 6). Furthermore, the homozygous subgroup had a significantly higher annual attack frequency than both the heterozygous and compound heterozygous subgroups (*p* < 0.001), (Table 6). In addition, the compound heterozygous subgroup had a significantly higher annual attack frequency than the heterozygous subgroup (*p* = 0.003), (Table 6).

Among the FMF patients, 31 (44.93%) carried the M694V variant, whereas 38 (55.07%) did not. Mean interleukin-6 levels were significantly higher in patients carrying the M694V variant than in those without the variant (58.99 ± 38.00 vs. 28.74 ± 37.45 pg/mL; B: 30.93 pg/mL, 95% CI: 10.69–51.18; *p* = 0.003). To evaluate whether the difference in interleukin-6 levels detected among *MEFV* mutation types was due to the M694V variant, interleukin-6 levels were compared in patients carrying and not carrying the M694V variant. Interleukin-6 levels in patients carrying and not carrying the M694V variant in *MEFV* mutation types are presented in Table 7.

Among *MEFV* gene mutation types, no significant difference was found in interleukin-6 levels according to M694V variant status in homozygous patients (Table 7), but the differences were borderline in heterozygous and compound heterozygous patients (Table 7).

ROC analysis was performed to evaluate the performance of interleukin-6 levels in discriminating clinical and genetic characteristics. The discriminatory performance of interleukin-6 levels between disease severity, colchicine resistance, and M694V variant status in the ROC analysis is presented in Table 8.

ROC analysis demonstrated that interleukin-6 levels could discriminate with high accuracy between moderate and mild disease severity, and between partial and non-collachicin-resistant states. The performance in discriminating between the positivity and negativity of the M694V variant was moderate (Table 8).

## 4. Discussion

FMF is one of the most common autoinflammatory illnesses, resulting from a congenital dysregulation of the immune system that leads to uncontrolled activation of inflammatory mediators and loss of function of regulatory mechanisms that limit inflammation. This condition causes an excessive inflammatory response to danger signals [23]. This pathophysiological process leads to the uncontrolled activation of inflammatory mediators and the inadequacy of regulatory mechanisms that suppress inflammation. As a result, serum values of various pro-inflammatory cytokines, particularly interleukin-6, and acute phase reactants are significantly elevated during FMF attacks [24]. However, while there is a general consensus in the literature that interleukin-6 levels are elevated during FMF attacks, different results have been reported regarding its clinical significance in the non-attack period. Oktem et al. A study reported that interleukin-6 values were significantly higher in adult FMF participants during attacks compared to healthy controls but remained within normal limits during non-attack periods and in heterozygous carriers, and therefore interleukin-6 was not sufficient to demonstrate subclinical inflammation [24]. In contrast, a study by Keskin et al. in adult FMF participants showed that serum interleukin-6 values were higher in both attack and non-attack periods compared to healthy controls [8].

This study found that interleukin-6 levels in blood samples taken during attack periods in pediatric FMF participants were higher compared to healthy children. Additionally, interleukin-6 levels were moderately positively correlated with the annual attack frequency in pediatric patients with FMF.

SAA is considered one of the most sensitive acute phase reactants in evaluating persistent subclinical inflammation in FMF patients and monitoring the effectiveness of colchicine treatment [25]. Persistently elevated SAA levels reflect ongoing systemic inflammation and are related with an increased risk of developing secondary amyloidosis [26]. Monitoring SAA values in FMF participants during non-attack periods is recommended to optimize colchicine treatment and minimize the risk of secondary amyloidosis. In countries where the SAA analysis is not routinely available, CRP, another acute phase reactant, is commonly used as an alternative. However, inconsistencies have been reported between these biomarkers; some patients may have high SAA levels despite normal CRP levels, while others may have the opposite [27]. A recent research in adult FMF participants demonstrated that normal CRP levels were associated with shorter time elapsed since the last attack, lower ISSF, non-M694V and low-penetrance mutations, and the need for increased colchicine doses. Conversely, persistently high CRP levels have been shown to be associated with M694V positivity, predominant serositis and musculoskeletal attacks, higher ISSF scores, colchicine resistance, treatment non-adherence, and increased frequency of emergency department visits [28].

This study found that SAA and CRP levels in blood samples taken during attack periods in pediatric FMF patients were significantly higher compared to healthy children. Additionally, strong positive correlations were found between interleukin-6 levels and SAA and CRP levels in pediatric FMF patients, whereas this relationship was not observed in healthy children. Furthermore, interleukin-6 levels were found to have a moderately positive correlation with ISSF score in pediatric FMF patients. Additionally, interleukin-6 levels were significantly higher in children with moderate FMF severity than in those with mild FMF severity according to the ISSF score. Additionally, interleukin-6 levels were significantly higher in children with FMF who had the M694V variant compared to children with FMF who did not have this variant.

In patients with FMF, similar to CRP, ESR levels have been reported to be significantly higher during attack periods compared to attack-free periods. Furthermore, ESR is considered a useful laboratory marker for assessing inflammation and indicating the presence of an attack in FMF [29]. A recent study in pediatric FMF patients showed that high ESR levels were significantly associated with colchicine resistance, and multivariate analysis revealed that the annual attacks frequency and high ESR levels were independent predictive factors for colchicine resistance. Additionally, it has been reported that ESR may be a useful biomarker in the early prediction of colchicine resistance in childhood FMF patients and may contribute to the development of appropriate treatment strategies through early detection of chronic inflammation [30]. A recent study conducted on pediatric FMF patients reported that, in addition to CRP and ESR, WBCs are also important parameters for distinguishing FMF patients from a healthy control group and for identifying inflammatory differences between attack and non-attack periods [31].

This study found that ESR and WBC levels in blood samples taken during attack periods in pediatric FMF participants were higher compared to healthy children. Additionally, interleukin-6 levels were significantly higher in children with partial colchicine resistance than in those without colchicine resistance. A strong positive correlation was found between interleukin-6 levels and ESR values in pediatric FMF patients, whereas this relationship was not observed in healthy children. On the other hand, a moderate positive relation was found between interleukin-6 levels and WBCs in healthy children, whereas this relationship was not observed in pediatric FMF patients. The moderate positive correlation between interleukin-6 and WBC levels observed in healthy children likely reflects the physiological role of interleukin-6 in regulating hematopoiesis and leukocyte homeostasis. In contrast, the absence of this correlation in pediatric FMF patients suggests that leukocytosis during FMF exacerbations is primarily influenced by interleukin-6 independent inflammatory pathways, particularly the activation of inflammasome-mediated innate immunity.

In a recent study of pediatric FMF patients, SAA, ESR, and WBC levels have been reported to be higher both during attack periods compared to non-attack periods, and in individuals with the M694V homozygous mutation compared to patients with other *MEFV* genotypes [31]. A recent experimental study using the M694I homozygous knock-in mouse model showed a significant increase in various pro-inflammatory cytokines, particularly serum interleukin-6 levels. Furthermore, the M694I homozygous variant was reported to be associated with marked cytokine dysregulation in these animals. These experimental findings support the idea that high-penetration mutations in the *MEFV* gene can potentiate the inflammatory response and that interleukin-6 plays a significant role in FMF pathophysiology [32]. However, in a research conducted on humans, patients were stratified into 3 groups according to their *MEFV* mutation status: M694I/E148Q compound heterozygous, M694I heterozygous, and E148Q heterozygous; no important difference in interleukin-6 levels was reported between these groups [33].

In this study, which compared subgroups based on *MEFV* mutation status, it was shown that interleukin-6 values were statistically importantly higher in participants with homozygous and compound heterozygous *MEFV* mutations compared to patients with heterozygous mutations; however, no important difference in interleukin-6 values was observed between the homozygous and compound heterozygous groups. Furthermore, the heterozygous subgroup had a significantly lower annual attack frequency than both the homozygous and compound heterozygous subgroups. Although the heterozygous subgroup had the largest sample size, no patients with partial colchicine resistance were identified in this subgroup. Consequently, the distribution of partial colchicine resistance differed significantly among the three *MEFV* mutation subgroups.

The heterozygous subgroup demonstrated a milder clinical phenotype than the homozygous and compound heterozygous subgroups, characterized by a lower annual attack frequency and the absence of colchicine resistance. These findings are consistent with the lower inflammatory burden observed in this subgroup and may partly explain the lower serum interleukin-6 levels. However, given the relatively small sample sizes of the mutation subgroups, these findings should be interpreted with caution and require confirmation in larger studies.

A recent study involving 869 children reported that the most common reason for requesting *MEFV* gene analysis was the presence of clinical symptoms consistent with FMF [34]. Another recent study showed that more pronounced clinical symptoms and longer disease duration were associated with M694V positivity and elevated CRP levels [28]. A study published this year demonstrated that a more severe disease course was associated with *MEFV* variants and that *MEFV* expression was linked to activation of the interleukin-6 signaling pathway. These findings support the importance of interleukin-6 in the pathogenesis of *MEFV*-related inflammation [35]. In parallel, a recent meta-analysis reported that among biological therapies used in colchicine-resistant or intolerant FMF patients, interleukin-6 inhibitors have an acceptable profile in terms of safety and efficacy, particularly in adult FMF participants [5].

This study found that pediatric FMF patients with symptoms such as abdominal pain, fever, arthralgia, or chest pain had higher interleukin-6 levels than those without these symptoms. Furthermore, a moderately positive correlation was found between the number of concomitant symptoms and interleukin-6 levels. Additionally, interleukin-6 levels were found to be higher in children with FMF who had 4 concomitant symptoms compared to those who had 1 or 2 symptoms. These results indicate that interleukin-6 levels may be related with clinical inflammatory burden and disease activity. Furthermore, a moderate positive correlation was found between interleukin-6 levels and disease duration, while no significant relationship was found between colchicine dose and interleukin-6 levels. The lack of a significant correlation between colchicine dose and interleukin-6 levels, together with the observed relationships between interleukin-6 levels and various clinical signs and disease duration, and the reported efficacy of interleukin-6 inhibitors in colchicine-resistant or colchicine-intolerant FMF participants, further supports the potential role of interleukin-6 as a biomarker of persistent inflammation and a therapeutic target in FMF.

The *MEFV* gene plays a critical role in the activation of the pyrin inflammasome in FMF. In FMF patients, *MEFV* expression in monocytes is variable and upregulated by pro-inflammatory cytokines, interferon-γ, tumor necrosis factor-α, lipopolysaccharide, and interleukin-1. Furthermore, *MEFV* gene mutations can lead to alterations in pyrin activity, affecting various responses of both innate and acquired immunity [36]. The pyrin inflammasome, activated by *MEFV* gene mutations, triggers the conversion of pro-interleukin-1β to active interleukin-1β via caspase-1 activation; the activation of the NF-κB signaling pathway by interleukin-1β via interleukin-1R1 can increase the production of various pro-inflammatory cytokines, including interleukin-6 [37].

This study found that patients with the M694V variant, which is associated with a more severe disease phenotype among *MEFV* gene mutations, had higher interleukin-6 levels. In contrast, no significant difference in interleukin-6 levels was found between patients carrying and not carrying the M694V variant within the *MEFV* mutation subgroups. On the other hand, ROC analysis showed that interleukin-6 has a moderate discriminatory ability in identifying M694V variant status. Furthermore, interleukin-6 was found to have a high discriminatory ability in determining disease severity (moderate–mild) and colchicine resistance (partial–absent) status.

A recent meta-analysis involving 96,153 participants showed that the *interleukin-6* rs1800795 polymorphism is associated with overall disease risk, particularly in Asian populations. Subgroup analyses revealed that this variant is associated with an increased risk of coronary artery disease, inflammatory diseases, and rheumatoid arthritis, while showing no significant association with type 1 diabetes, type 2 diabetes, and some other diseases [38]. A meta-analysis encompassing different regional populations has shown that the effect of the *interleukin-6* rs1800795 polymorphism on tuberculosis susceptibility may vary according to ethnicity, and this relationship is particularly significant in individuals of Asian descent [39]. A meta-analysis encompassing diverse populations has shown that the *interleukin-6* rs1800795 polymorphism is moderately associated with obesity risk, but that various factors, including environmental factors, play a role in the development of obesity [40]. Consistent with the heterogeneous findings reported across different diseases, a recent meta-analysis demonstrated no significant association between the *interleukin-6* rs1800795 polymorphism and susceptibility to depression under any genetic model, indicating that the contribution of this variant may be disease-specific and influenced by gene–environment interactions [41].

This research compared the allele and genotype distributions of the *interleukin-6* rs1800795 variant between a Turkish cohort of childhood FMF participants and healthy children and found no important difference between the groups. Furthermore, no important difference was found in interleukin-6 levels among pediatric FMF patients with different genotypes of the *interleukin-6* rs1800795 variant. However, the fact that the rs1800795 CC genotype was found in only one participant in both the FMF and control groups significantly limits the study’s power to detect small and medium-scale genetic effects. Therefore, the current findings suggest no significant association between the rs1800795 variant and FMF susceptibility or inflammatory activity, but this does not definitively prove the absence of such an association. Moreover, due to the limited sample size and the variability in genetic variations in different ethnic populations, these findings need to be confirmed by research including different ethnic groups and larger sample sizes.

In this study, healthy controls were not genotyped for *MEFV* variants; therefore, the presence of asymptomatic *MEFV* variant carriers in the control group cannot be ruled out. Although controls underwent clinical and laboratory screening to rule out overt inflammatory, infectious, autoimmune, and autoinflammatory conditions, subclinical inflammation or undetected autoinflammatory disease may still be present. Furthermore, the lack of a disease control group comprising patients with other recurrent fevers or autoinflammatory disorders limits the specificity of the findings to the disease. Additionally, the genetic heterogeneity of the *MEFV* mutation spectrum and the relatively small number of patients within individual mutation subgroups limited our ability to perform mutation-specific association analyses. Consequently, the observed increase in interleukin-6 levels cannot be interpreted as specific to FMF and may partially reflect a general inflammatory response. Future prospective studies including both healthy controls and disease-control groups with other autoinflammatory disorders are needed to determine whether the observed increase in interleukin-6 is specific to FMF.

One limitation of this study is that samples from the patient group were collected only during acute attacks. While this design allows for the assessment of differences between patient and control groups, it prevents determining whether interleukin-6 reflects ongoing subclinical inflammation during remission. Therefore, the current findings do not allow for distinguishing whether interleukin-6 is merely a marker reflecting acute inflammation or a biomarker indicating chronic disease activity. Another limitation is that although the sample size appears sufficient to detect differences in circulating interleukin-6 levels, it is relatively small for a genetic association study. The single-center design and the inability to measure other important cytokines involved in FMF pathogenesis (e.g., interleukin-1β and interleukin-18) can also be identified as other significant limitations. On the other hand, one of the strengths of this study is that it serves as a reference for future multi-center studies where these limitations can be overcome.

## 5. Conclusions

In this study, higher interleukin-6 levels were observed in pediatric FMF patients compared to healthy children. Interleukin-6 levels showed a positive correlation with SAA, CRP, ESR, ISSF score, annual attack frequency, the number of concomitant symptoms and disease duration, and they differed according to clinical symptoms and *MEFV* mutation status. Additionally, interleukin-6 levels were higher in children with moderate FMF severity than in those with mild FMF severity. Moreover, interleukin-6 levels were found to be higher in children with FMF who had 4 concomitant symptoms compared to those who had 1 or 2 symptoms. Similarly, interleukin-6 levels were higher in children with partial colchicine resistance than in those without colchicine resistance. Additionally, children carrying the M694V variant had higher interleukin-6 levels compared to children without this variant. Interleukin-6 has a moderate ability to determine M694V variant status, while it has a high level of discriminatory ability in determining disease severity (moderate–mild) and colchicine resistance (partial–absent). In contrast, no significant relationship was observed between interleukin-6 levels and colchicine dose. Furthermore, no significant difference was found between pediatric FMF patients and healthy controls in terms of allele and genotype distributions of the *interleukin-6* rs1800795 polymorphism, and interleukin-6 levels did not differ among rs1800795 genotypes. While these findings suggest that the *interleukin-6* rs1800795 polymorphism is unlikely to be associated with genetic predisposition to FMF or inflammatory activity in this cohort, the limitations of sample size for genetic assessment should not be disregarded. However, the findings should be interpreted considering the study’s limitations, such as the single-center design, the relatively small sample size, and the fact that all blood samples were collected during acute FMF attacks. Therefore, these results may not be generalizable to patients in the relapsed period or to other populations. Larger prospective multicenter studies including various ethnic groups are needed to confirm these findings and further clarify the clinical significance of interleukin-6 and its genetic variants in pediatric FMF.

## Figures and Tables

**Table 1:** Laboratory parameters of the control and FMF groups.

Parameters	Control Group (*n* = 50)	FMF Group (*n* = 69)	*p*
Interleukin-6 (pg/mL)	3.43 ± 1.65	42.33 ± 40.37	<0.001
Serum amyloid A (mcg/mL)	0.66 ± 0.33	4.45 ± 5.72	0.001
C-reactive protein (mg/L)	2.54 ± 1.05	15.66 ± 19.44	0.027
Erythrocyte sedimentation rate (mm/hr)	3.5 ± 1.63	13.28 ± 10.88	<0.001
White blood cell count (10^3^/μL)	7.33 ± 1.73	9.8 ± 3.38	<0.001
Neutrophil count (10^3^/μL)	4.32 ± 1.61	5.81 ± 3.24	0.028
Lymphocyte count (10^3^/μL)	3.01 ± 0.85	3.25 ± 1.41	0.667
NLR	1.52 ± 0.72	2.3 ± 2.4	0.166
Hemoglobin (g/dL)	11.78 ± 1.58	12.3 ± 1.21	0.080
Platelet count (10^3^/μL)	360 ± 90.43	379.24 ± 120.5	0.445

FMF: Familial Mediterranean Fever.

**Table 2:** Correlation analysis between interleukin-6 levels and laboratory parameters in FMF and control groups.

Parameters		Control Group (*n* = 50)	FMF Group (*n* = 69)
		Interleukin-6	Interleukin-6
Serum amyloid A	ρ	−0.033	0.838
	*p*	0.818	<0.001
C-reactive protein	ρ	−0.155	0.888
	*p*	0.282	<0.001
Erythrocyte sedimentation rate	ρ	0.022	0.888
	*p*	0.877	<0.001
White blood cell count	ρ	0.405	0.086
	*p*	0.004	0.482
Neutrophil count	ρ	0.275	0.064
	*p*	0.054	0.604

FMF: Familial Mediterranean Fever, ρ: Spearman correlation coefficient.

**Table 3:** Distribution of *interleukin-6* rs1800795 (-174 G>C) gene variant in FMF and control groups.

*Interleukin-6* rs1800795 Variant		Control Group (*n* = 50)	FMF Group (*n* = 69)	*p*
		HWE *p* = 0.377	HWE *p* = 0.171	
Genotype *n* (%)	GG	31 (62)	42 (60.87)	0.961
	GC	18 (36)	26 (37.68)	
	CC	1 (2)	1 (1.44)	
Allele *n* (%)	G	80 (80)	110 (79.71)	0.941
	C	20 (20)	28 (20.29)	

FMF: Familial Mediterranean Fever, HWE: Hardy–Weinberg equilibrium.

**Table 4:** Interleukin-6 levels in the FMF group patients according to the presence of symptoms, colchicine resistance, and ISSF severity categories.

			FMF Group (*n* = 69)		*p*	B (95% CI)
			*n* (%)	Interleukin-6 (pg/mL)		
Symptoms	Abdominal pain	Yes	51 (73.91)	52.23 ± 41.03	0.001	38.119 (16.644–59.594)
		No	18 (26.09)	14.28 ± 20.98		
	Fever	Yes	56 (81.16)	47.84 ± 41.51	0.023	29.515 (4.261–54.769)
		No	13 (18.84)	18.59 ± 24.21		
	Chest pain	Yes	33 (47.83)	52.27 ± 43.53	0.002	29.059 (10.626–47.493)
		No	36 (52.17)	28.63 ± 32.10		
	Arthralgia	Yes	33 (47.83)	61.65 ± 43.22	<0.001	37.185 (19.119–55.251)
		No	36 (52.17)	24.62 ± 28.01		
Colchicine resistance		Partial	13 (18.84)	90 ± 29.46	<0.001	59.899 (39.087–80.711)
		No	56 (81.16)	31.26 ± 34.11		
ISSF severity categories		Mild	51 (73.91)	27.13 ± 31.59	<0.001	59.774 (41.435–78.113)
		Moderate	18 (26.09)	85.39 ± 30.46		

FMF: Familial Mediterranean Fever, B: Adjusted Mean Difference, CI: Confidence Interval, ISSF: International Severity Scoring System for Familial Mediterranean Fever.

**Table 5:** Distribution of *MEFV* genotype categories among in the FMF group (*n* = 69).

Genotype Category	*MEFV* Genotype(s)	*n* (%)
Homozygous pathogenic	M694V/M694V, M680I/M680I, M694I/M694I	14 (20.29)
Heterozygous pathogenic	M694V, M680I, M694I, V726A,	26 (37.68)
Homozygous VUS	E148Q/E148Q	2 (2.90)
Heterozygous VUS	E148Q	10 (14.49)
Compound heterozygous	M694V/E148Q, M694V/M680I, M694V/M694I, M694V/V726A, M694V/K695R, M694I/R761H, V726A/E148Q, M680I/V726A, E148Q/P369S	17 (24.64)
Total		69 (100)

VUS: Variant of uncertain significance.

**Table 6:** Interleukin-6 levels according to *MEFV* gene mutation types in the FMF group.

Parameters		Homozygous (*n* = 16)	Heterozygous (*n* = 36)	Compound Heterozygous (*n* = 17)	*p*
Interleukin-6 (pg/mL)		74.16 ± 39.30	23.18 ± 27.41	52.92 ± 43.34	<0.001
Annual attacks frequency		4.5 ± 0.89	1.86 ± 0.68	2.76 ± 1.25	<0.001
Age of diagnosis		6.25 ± 3.90	7.6 ± 4.22	6.62 ± 3.91	0.486
Colchicine resistance	Partial	10 (62.5)	0 (0)	3 (17.65)	<0.001
	No	6 (37.5)	36 (100)	14 (82.35)	

**Table 7:** Interleukin-6 levels in patients carrying and not carrying the M694V variant in *MEFV* gene mutation types.

*MEFV* Gene Mutation Types	M694V Variant	*n* (%)	Interleukin-6 (pg/mL)	*p*
Homozygous (*n* = 16)	Yes	10 (62.5)	77 ± 29.95	0.828
	No	6 (37.5)	69.42 ± 54.54	
Heterozygous (*n* = 36)	Yes	10 (27.78)	29.27 ± 22.86	0.059
	No	26 (72.22)	20.84 ± 29.04	
Compound heterozygous (*n* = 17)	Yes	11 (64.71)	69.63 ± 41.49	0.050
	No	6 (35.29)	22.28 ± 28.74	

**Table 8:** Diagnostic performance of interleukin-6 levels for discriminating disease severity, colchicine resistance, and M694V variant status.

	Disease Severity	Colchicine Resistance	M694V Variant
Subgroup	Moderate	Partial	+
Reference	Mild	Absent	−
AUC (95% CI)	0.883 (0.805–0.961)	0.883 (0.802–0.965)	0.741 (0.622–0.859)
Cut Off	57.50	66	33
*p*	<0.001	<0.001	0.001
Sensitivity (%)	83.30	76.90	71
Specificity (%)	80.40	76.80	71.10
PPV (%) (95% CI)	60 (38.67–78.87)	43.48 (23.19–65.51)	66.70 (49.60–80.30)
NPV (%) (95% CI)	93.18 (81.34–98.57)	93.48 (82.1–98.63)	75 (58.9–86.2)

AUC: Area under curve, CI: Confidence interval PPV: Positive predictive value, NPV: Negative predictive value.

## Data Availability

The datasets generated and analyzed during the current study are not publicly available due to patient privacy regulations but are available from the corresponding author upon reasonable request and with appropriate ethical approvals.

## Abbreviations

The following abbreviations are used in this manuscript:

CRP	C-reactive protein
ESR	Erythrocyte sedimentation rate
ISSF	International Severity Scoring System for Familial Mediterranean Fever
FMF	Familial Mediterranean fever
*MEFV*	Mediterranean fever gene
PCR–RFLP	Polymerase chain reaction–restriction fragment length polymorphism
PRINTO	Eurofever/Pediatric Rheumatology International Trials Organization
SAA	Serum amyloid A
WBC	White blood cell count
